# Supplementary material for: White LED intensities during co-cultivation affect the Agrobacterium-mediated soybean (Glycine max) transformation using mature half seeds as explants
Source: PLoS One. 2024 Nov 26;19(11):e0312129. doi: 10.1371/journal.pone.0312129 (PMC11594425; doi:10.1371/journal.pone.0312129)
Supplement: S1 File — (DOCX) [file pone.0312129.s001.docx]

**Supporting information**

**S1 Table. Inhibitory effects of various concentration of glufosinate on soybean ‘Thorne’ half-seed explant shoot induction.** The inhibition percentage is calculated as $\frac{number of explants without shoot}{number of total explants}\times100$. Data is present as mean ± standard deviation (n = 18).

| Glufosinate concentration (mg/L) | Inhibition percentage (%) ^*^ |
| --- | --- |
| 0 | 0 |
| 2 | 46.7 ± 19.6 |
| 4 | 86.7 ± 10.9 |
| 6 | 100 ± 0 |
| 8 | 100 ± 0 |

^*^: The inhibition percentage is normalized by subtracting shoot induction percentage at 0 mg/L of glufosinate.

**S2 Table. Transformation efficiencies under tembotrione selection after various light intensities during co-cultivation.** Values are present as mean $\pm$ standard error (n = 6).

| **Light Source** | **Light Intensity**  **(**$\boldsymbol{\mu mol\cdot}\mathbf{m}^{\mathbf{-2}}\boldsymbol{\cdot}\mathbf{s}^{\mathbf{-1}}$**)** | **Transformation Efficiency ^ns^**  **(%)** |
| --- | --- | --- |
| White LED | 50 | 2.4 $\pm$ 0.9 |
|  | 100 | 2.5 $\pm$ 1.3 |
|  | 150 | 4.5 $\pm$ 1.1 |
|  | 190 | 5.2 $\pm$ 1.2 |
| Fluorescent | 100 | 5.4 $\pm$ 1.5 |
